# Social support rescues acute stress-induced cognitive impairments by modulating ERK1/2 phosphorylation in adolescent mice

**DOI:** 10.1038/s41598-018-30524-4

**Published:** 2018-08-13

**Authors:** Ji-Woon Kim, Mee Jung Ko, Edson Luck Gonzales, Ri Jin Kang, Do Gyeong Kim, Yujeong Kim, Hana Seung, Hyun A Oh, Pyeong Hwa Eun, Chan Young Shin

**Affiliations:** 1 Department of Neuroscience, School of Medicine, Konkuk University, Seoul, 143-701 Korea; 2 Department of Advanced Translational Medicine, Konkuk University, Seoul, 143-701 Korea; 3 Department of Pharmacology, School of Medicine, Konkuk University, Seoul, 143-701 Korea

## Abstract

Social support can relieve stress-induced behavioural outcomes, although its underlying molecular mechanisms are not fully understood. Here, we evaluated whether social interactions can prevent the restraint stress (RS)-induced cognitive impairments in male adolescent mice by utilizing molecular, cellular, and behavioural approaches. Acute RS in adolescent ICR mice impaired the working memory in the Y-maze test and memory consolidation and retrieval in the novel-object-recognition test (NORT). In addition, RS increased the extracellular signal-regulated kinases 1/2 phosphorylation (p-ERK1/2) in the prefrontal cortex (PFC) and corticosterone levels in the plasma. Interestingly, these outcomes were normalized by the presence of a conspecific animal (social support) during RS. RS also significantly upregulated the expression levels of known stress-relevant genes such as *Egr1*, *Crh*, *and Crhr1*, which were normalized by social support. Systemic injection of SL327 (an inhibitor of MEK1/2 that also blocks its downstream signal ERK1/2) prior to RS rescued the working memory impairments and the increased p-ERK1/2 while normalizing the expression of *Egr1*. Our results suggest that social support can alleviate the RS-induced cognitive impairments partly by modulating ERK1/2 phosphorylation and gene transcription in the PFC, and provide novel insights into the molecular mechanisms of the stress-buffering effects of social support.

## Introduction

Stress can significantly impact the psychological, neurological and physiological functions, which can lead to health problems such as psychiatric disorders, immunosuppression, and cardiovascular diseases^1–3^. Adolescents are especially vulnerable to traumatic stress and often display exaggerated stress responses compared to adults because of the maturational changes in cortico-limbic regions and HPA axis^4,5^. In addition, steroid hormones can affect the juvenile brain reorganizations and neural circuits^6^. Thus, uncontrolled stress during this period may potentially impact brain development and cause serious neuropsychiatric disorders including mood disorders^7^, memory disorders, and post-traumatic stress disorder in their adulthood^8,9^. Thereby, it is imperative to develop effective coping strategies against stress-induced outcomes during adolescence.

Social support has been suggested as an effective coping strategy to relieve stress-induced behavioural changes^10,11^. Researchers have found that social support in humans could reduce stress hormone levels as well as prevent depressive behaviours brought by stressful conditions^10,12,13^. Social support could also provide positive outcomes for individuals who experienced early life stresses like child abuse, stigma, daily hassles, major life events or war in severe cases^14–16^. In animals, particularly guinea pigs and prairie voles, social support can facilitate the recovery of depressive-like behaviours and increased cortisol levels induced by stress^17,18^. Moreover, the increments of cortisol in isolated marmoset monkeys were relieved by exposure to conspecific vocalizations^19,20^. However, little is known about the molecular mechanisms involved in the stress-buffering effects of social support, which we investigated in the current study.

Acute restraint stress (RS) is an established model known to induce various changes in behaviour and expression of neurochemicals. Acute restraint stress can rapidly change the catecholamine levels in the brain, such as dopamine and norepinephrine, and activate the hypothalamic-pituitary-adrenocortical axis (HPA), which are followed by increments of stress hormones^21^. Subsequently, these stress-induced responses activate various signalling molecules such as ERK1/2, a member of the mitogen-activated protein kinases (MAPK), majorly in the prefrontal cortex (PFC) and hippocampus^22–24^. Stress also modulates neuronal plasticity by altering gene transcription and neural activity^25,26^. These collective changes can affect brain functions, especially cognitive processes such as novel object recognition^27^, reversal learning^28^, and retrieval of long-term memory^29^.

Here, we investigated whether social support during acute restraint stress can relieve the stress-induced cognitive deficits and molecular changes in adolescent mice. We characterized the changes in cognitive function using Y-maze test and novel object recognition test, and measured the level of ERK1/2 phosphorylation in the PFC and corticosterone in the plasma. We then utilized a brain-penetrable MEK1/2 (an upstream molecule of ERK1/2) inhibitor, SL327, to block the stress-induced ERK1/2 phosphorylation and investigated the role of ERK1/2 in stress-induced cognitive deficits with Y-maze test. Lastly, we measured the transcriptional changes to confirm the social support effects on the stress-induced gene expression changes, which might continuously affect the neural plasticity and stress response. Our findings demonstrate the positive effects of social support against acute restraint stress and the role of ERK1/2 phosphorylation in the alleviation of stress-induced cognitive deficits during the adolescent period.

## Results

### Social interaction improved the working memory impairments in restraint-stressed adolescent mice

**Figure 1:**
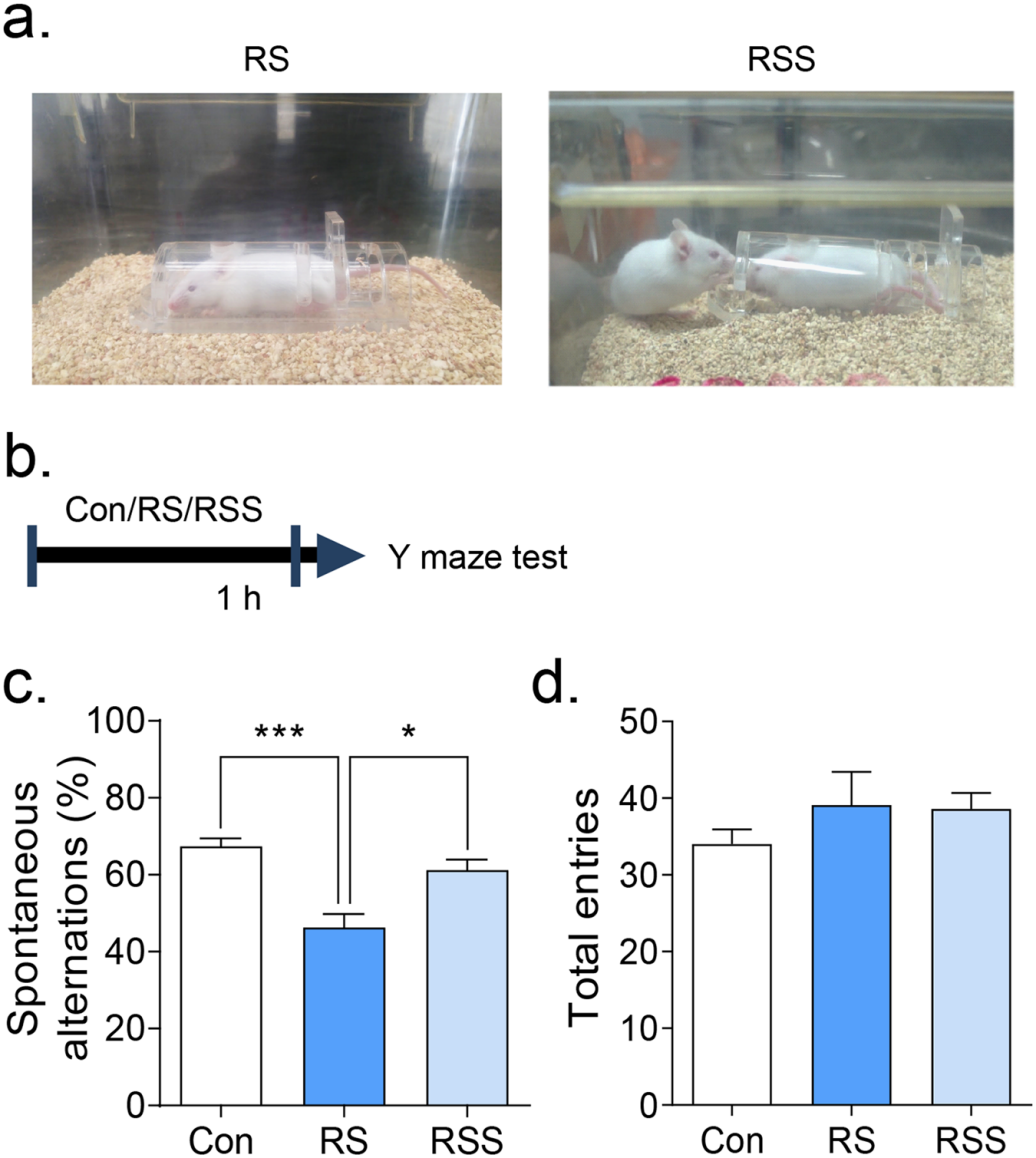
Social interaction rescued the restraint stress-induced working memory impairments. (**a**) Images representing restraint stress (RS) and restraint stress with the presence of conspecific mouse (RSS) conditions. (**b**) Scheme of Y maze test performed after an hour of RS or RSS. (**c**) Spontaneous alternations and (**d**) total arm entries were evaluated in the Y maze test (n = 10). All data are expressed as the mean ± S.E.M using bar graphs. (**c**) one-way ANOVA followed by Bonferroni’s multiple comparison post hoc analysis and (**d**) Kruskal-Wallis test followed by Dunn’s multiple comparison post hoc analysis. * is *p* < 0.05, *** is *p* < 0.001. Con: vehicle control group, RS: restraint stress group, RSS: restraint stress with social interaction group.

To investigate the role of social support during stress, we adopted a prosocial behaviour paradigm^30^ (Fig. 1a). After an hour of restraint stress, the animals’ capability to acquire working memory derived from their willingness to explore new environments were evaluated by Y-maze test (Fig. 1b–d), which is expressed by spontaneous alternation of arm entries (Fig. 1c). There was no statistical difference observed in the total arm entries of each group (Fig. 1d, F(2, 27) = 0.8879, *p* = 0.4232), showing no difference in locomotor activity. However, the spontaneous alternation score was decreased in the restraint stress group compared to the control group (Fig. 1c, H = 15.67, p < 0.001). Interestingly, the presence and interaction of conspecific mice with restrained mice (RSS) during RS improved the working memory deficits of the stressed mice (p < 0.05 vs RS; no significance vs Con). To investigate whether these effects were in fact induced from the social interaction with a novel conspecific mouse, we put a novel object (stacked Lego blocks) instead of a conspecific mouse during restraint stress in a separate experiment (Supplementary Fig. 1). We found that presence of a novel object did not rescue the reduced spontaneous alternations of RS mice, suggesting that the rescuing effects of social support are induced by the social interaction (Supplementary Fig. 1b).

Additionally, we investigated the social support effects on the RS-induced anxiety-like behaviours in the elevated plus maze test (EPM) and open field test (OFT) (Supplementary Fig. 2). In the EPM, RS and RSS groups showed slightly but not significantly reduced stay duration and movement in open arms over closed arms (Supplementary Fig. 2a, H = 2.611, p = 0.2710, and 2b, H = 2.01, p = 0.3660). In the OFT, RS and RSS groups also did not show any differences of general movements in the whole arena or in the centre area compared to control (Supplementary Fig. 2c, F(2, 27) = 0.2138, p = 0.8089, and 2d, F(2, 27) = 0.5666, p = 0.5741). Thus, in our study condition, RS did not significantly induce anxiety-like phenotypes.

### Recognition impairments were improved by social interaction in restraint-stressed adolescent mice

**Figure 2:**
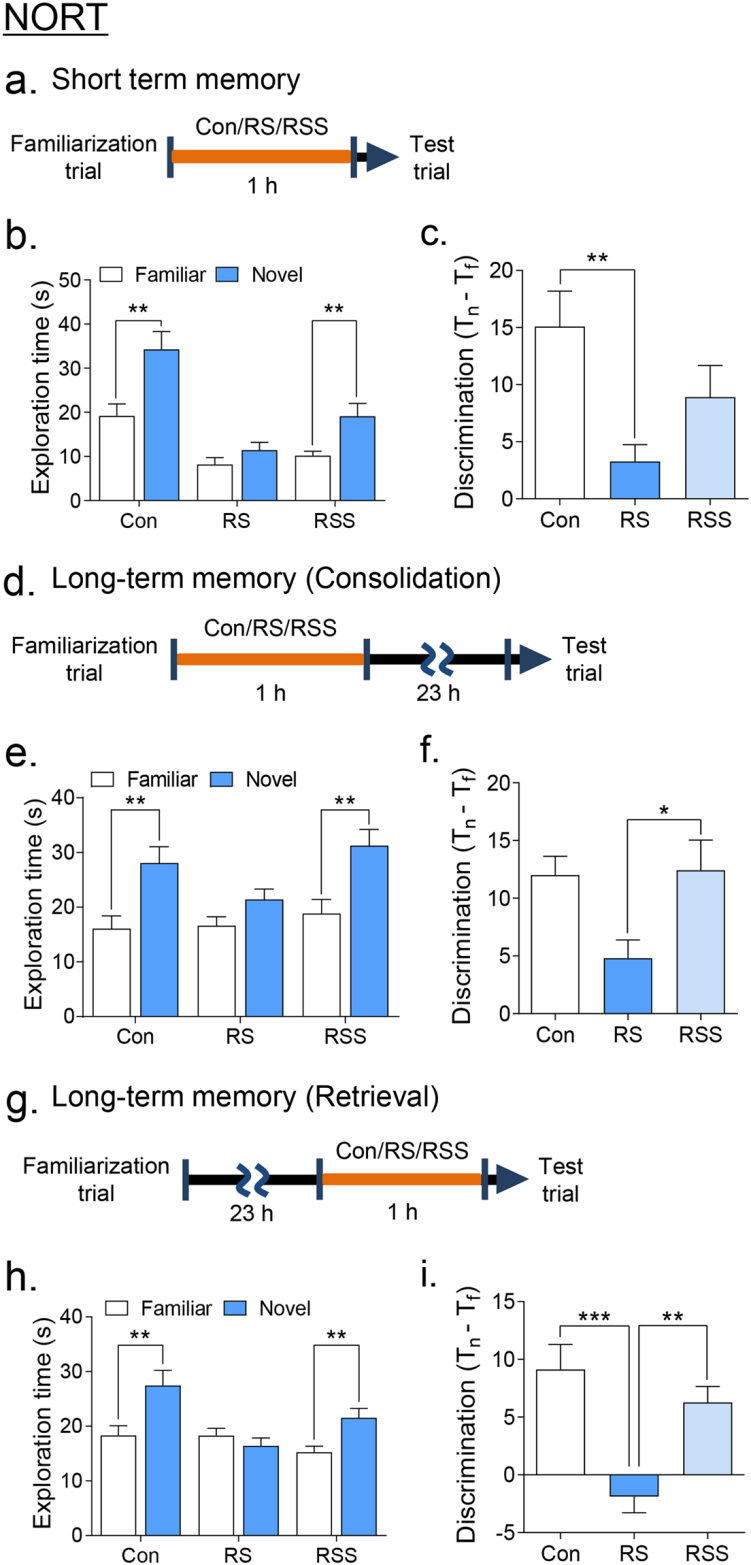
Social interaction rescued the restraint stress-induced object recognition impairments. Schedule of NORT (**a**) short-term memory and long-term memory paradigms at the (**d**) consolidation and (**g**) retrieval phases of restraint application. (**b**,**e**,**h**) Exploration times in the familiar and novel objects. (**c**,**f**,**i**) Discrimination values calculated as the difference in exploration time between novel (T_n_) and familiar object (T_f_). All data are expressed as the mean ± S.E.M using bar graphs. (**b**,**e**,**h**) Unpaired t-test or Mann Whitney test to compare the exploration time between familiar and novel object. (**c**,**f**,**i**) one-way ANOVA of discrimination values followed by Bonferroni’s multiple comparison post hoc analysis. ((**b**,**c**) n = 11, (**e**,**f**) n = 10–12, (**h**,**i**) n = 11–12). * is *p* < 0.05, ** is *p* < 0.01, *** is *p* < 0.001. Con: vehicle control group, RS: restraint stress group, RSS: restraint stress with social interaction group.

To evaluate whether acute RS can affect the short-term or long-term memory during object recognition in mice^27^, we restrained the mice in between the familiarization and novel object recognition trials. In the short-term memory paradigm (Fig. 2a), we identified that acute restraint stress affected the exploration time in the novel versus familiar objects showing no significant recognition of the novel object (Fig. 2b, p > 0.05), unlike control mice (p < 0.01, novel vs familiar). Remarkably, the RSS group showed significantly improved recognition of the novel object (Fig. 2b, p < 0.01, novel vs familiar). In addition, the calculation for discrimination (exploration time difference between novel objects (T_n_) and familiar objects (T_f_)) showed significantly lowered discrimination in RS group compared to Con group (p < 0.01) but not between RSS vs Con. There was only a tendency of increased discrimination in RS vs RSS.

Of further interest in our results, the consolidation (Fig. 2d–f) and retrieval (Fig. 2g–i) of long-term memory were also affected by restraint stress in the NORT. The mice given RS at the consolidation stage failed to significantly recognize the novel object based on exploration time (Fig. 2e, p > 0.05) whereas RSS group mice exhibited a normalized exploration time around the novel object (Fig. 2e, p < 0.01). However, the calculation for discrimination showed a significant difference between RS and RSS groups only (Fig. 2f, F(2, 30) = 4.014, p = 0.0285). More evident recovery of impaired recognition was observed in the mice given RS at the retrieval stage. The presence of social support also rescued the impaired novel object recognition in the RS group (Fig. 2h, p > 0.05). The decreased discrimination value in the RS group was also normalized to the level of the control group in the RSS group (Fig. 2i, F(2, 32) = 11.89, p = 0.0001). Collectively, restraint stress can affect both the short-term and long-term memory processes during the consolidation and retrieval stages as previously described^27^, whereas the presence of a social support could alleviate these recognition impairments.

### Social interaction reduced the ERK1/2 phosphorylation induced by acute restraint stress in the PFC and normalized the plasma corticosterone levels

**Figure 3:**
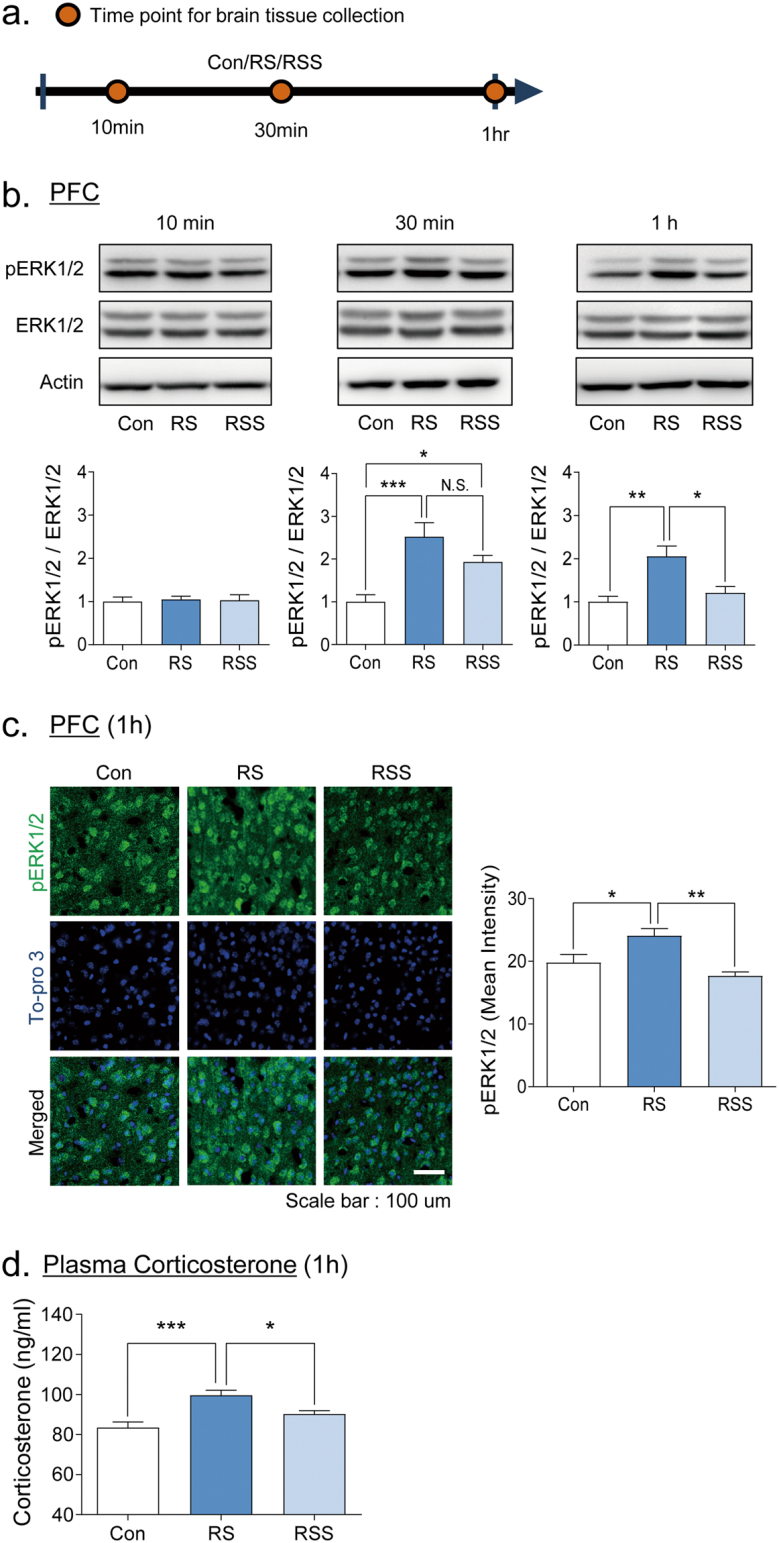
Restraint-stress induced ERK1/2 activation in the prefrontal cortex is normalized by social interaction. (**a**) Scheme of brain tissue collection at three different restraint stress durations. The activation of ERK1/2 was measured in the prefrontal cortex after acute restraint stress using western blot (**b**) and immunohistochemistry (**c**). Western blot representative images cropped from separate sets of gels per time point and the corresponding quantitative graphs of ERK1/2-phosphorylation divided by ERK1/2 bands intensity (n = 5–6). Quantifications of pERK1/2/ERK1/2 bands were presented as the fold change normalized by band intensity of control group. ERK1/2 phosphorylation was significantly increased by 30 min or 1 h of restraint stress. (**c**) Immunohistochemistry images of layer 4 to 5 medial PFC and quantitative graph (n = 5). (**d**) Plasma corticosterone levels after 1 h of RS or RSS. All data are expressed as the mean ± S.E.M using bar graphs. All statistical analyses were performed using one-way ANOVA and Bonferroni’s multiple comparison post hoc analysis. *p < 0.05, **p < 0.01, ***p < 0.001. N.S.: no significance. Con: vehicle control group, RS: restraint stress group, RSS: restraint stress with social interaction group.

Previous studies have reported that ERK1/2 phosphorylation is triggered by acute restraint stress in the PFC and hippocampus regions^22,23^. Therefore, we evaluated the levels of ERK1/2 phosphorylation after 10 min, 30 min and 1 h of acute restraint stress in the PFC, hippocampus, and amygdala (Fig. 3a and Supplementary Fig. 3). We found that ERK1/2 phosphorylation was significantly increased in mice with restraint stress for 30 min and 1 h in the prefrontal cortex region (Fig. 3b). Interestingly, the ERK1/2 phosphorylation of 1 h restraint-stressed mice was significantly reduced when they were accompanied by a conspecific stranger mouse (Fig. 3b, 30 min: F(2, 15) = 11.13, *p* < 0.01, 1 h: F(2, 15) = 10.65, *p* < 0.01). To investigate the region-specific ERK1/2 phosphorylation activity, we stained phosphor-ERK1/2 in the medial prefrontal cortex, specifically layer 4–5, with immunohistochemistry (Fig. 3c). We found that 1 h restraint stress increased the intensity of ERK1/2 phosphorylation whereas the presence of a conspecific stranger mouse during restraint stress prevented its increase (F(2, 12) = 9.294, *p* < 0.01). In the hippocampus, significant up-regulation of ERK1/2 phosphorylation was observed only in the 1-h restraint stress group, which was recovered in restrained mice with the presence of a conspecific mouse (F(2, 12) = 9.238, *p* < 0.01, Supplementary Fig. 3a). No changes in ERK1/2 phosphorylation were observed in the amygdala region (Supplementary Fig. 3b).

We also measured the corticosterone levels in the plasma to confirm the stress hormonal changes at 1 h of RS and RSS. Interestingly, the RS group showed a significant increase of corticosterone whereas the RSS group was comparable to the levels of the control group, suggesting that 1 h of social support is enough to relieve the stress-induced hormonal changes (H = 18.26, p = 0.0001).

### Inhibition of ERK1/2 phosphorylation by SL327 ameliorated the cognitive impairments induced by an hour of restraint stress

**Figure 4:**
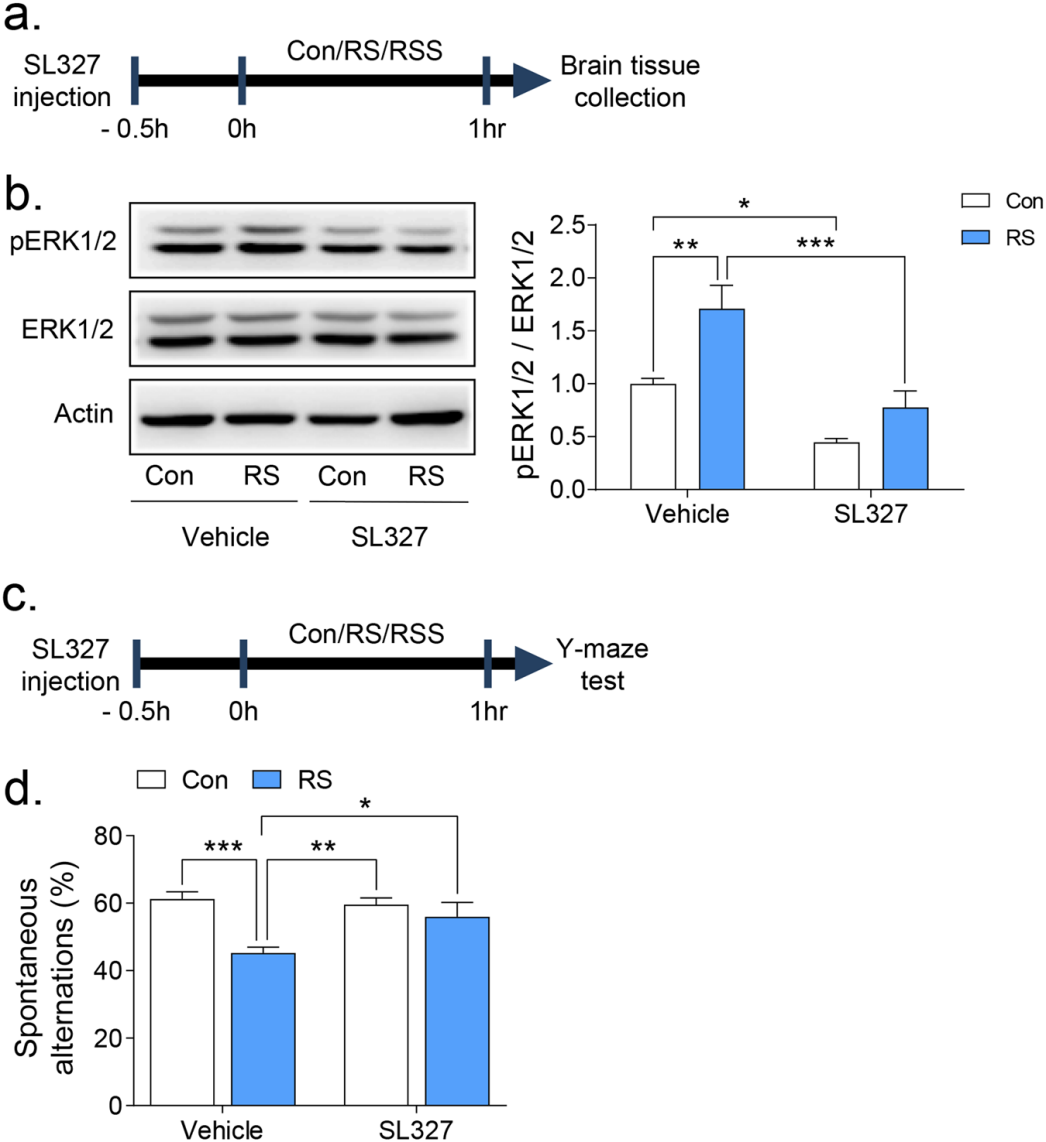
Inhibition of ERK1/2 phosphorylation rescued the restraint stress-induced working memory impairment. A brain penetrant ERK1/2 inhibitor, SL327, was injected i.p. 30 min before restraint stress followed by brain preparation (**a**,**b**) or Y maze test (**c**,**d**). (**b**) Western blot representative images and the graphical representation of pERK1/2 divided by ERK1/2 bands intensity after SL327 treatment and restraint stress (n = 5). Quantifications of pERK1/2/ERK1/2 were presented as the fold change normalized by control. (**d**) Y-maze test showing the spontaneous alterations of each group (n = 12). All data are expressed as the mean ± S.E.M using bar graphs. All statistical analyses were performed using two-way ANOVA test and Bonferroni’s multiple comparison post hoc analysis. * is *p* < 0.05, ** is *p* < 0.01, *** is *p* < 0.001. Con: vehicle control group, RS: restraint stress group.

So far, we found that social interaction could normalize the upregulated ERK1/2 phosphorylation and corticosterone levels, as well as the impaired cognitive functions in mice given a restraint stress. We then investigated the role of ERK1/2 in the stress-buffering effects of social interaction. To address this question, we utilized SL327, a highly selective and brain penetrable MEK1/2 inhibitor to block ERK1/2^31,32^ (Fig. 4a). In the Western blot analysis, systemic administration of SL327 ablated the previously enhanced ERK1/2 phosphorylation in the prefrontal cortex of mice after 1 h restraint stress (Fig. 4b, Restraint × Drug effect: F(1, 16) = 1.88, *p* = 0.189, Restraint effect: F(1, 16) = 14.11, *p* < 0.01, Drug effect: F(1, 16) = 28.84, *p* < 0.0001). Next, we investigated whether SL327 reverses the stress-induced working memory impairments in the Y-maze test (Fig. 4c). Interestingly, the reduced spontaneous alternation in the RS group was significantly increased by SL327 administration (Fig. 4d, Restraint × Drug effect: F(1, 40) = 5.26, *p* < 0.05, Restraint effect: F(1, 40) = 13.09, *p* < 0.001, Drug effect: F(1, 40) = 2.82, *p* = 0.1008). Our findings suggest that modulation of ERK1/2 phosphorylation may mediate the acute stress-induced cognitive impairments as well as the stress-buffering effects of social support.

### Social interaction and SL327 modulated the mRNA transcription levels of several stress-related genes during restraint stress in mice

**Figure 5:**
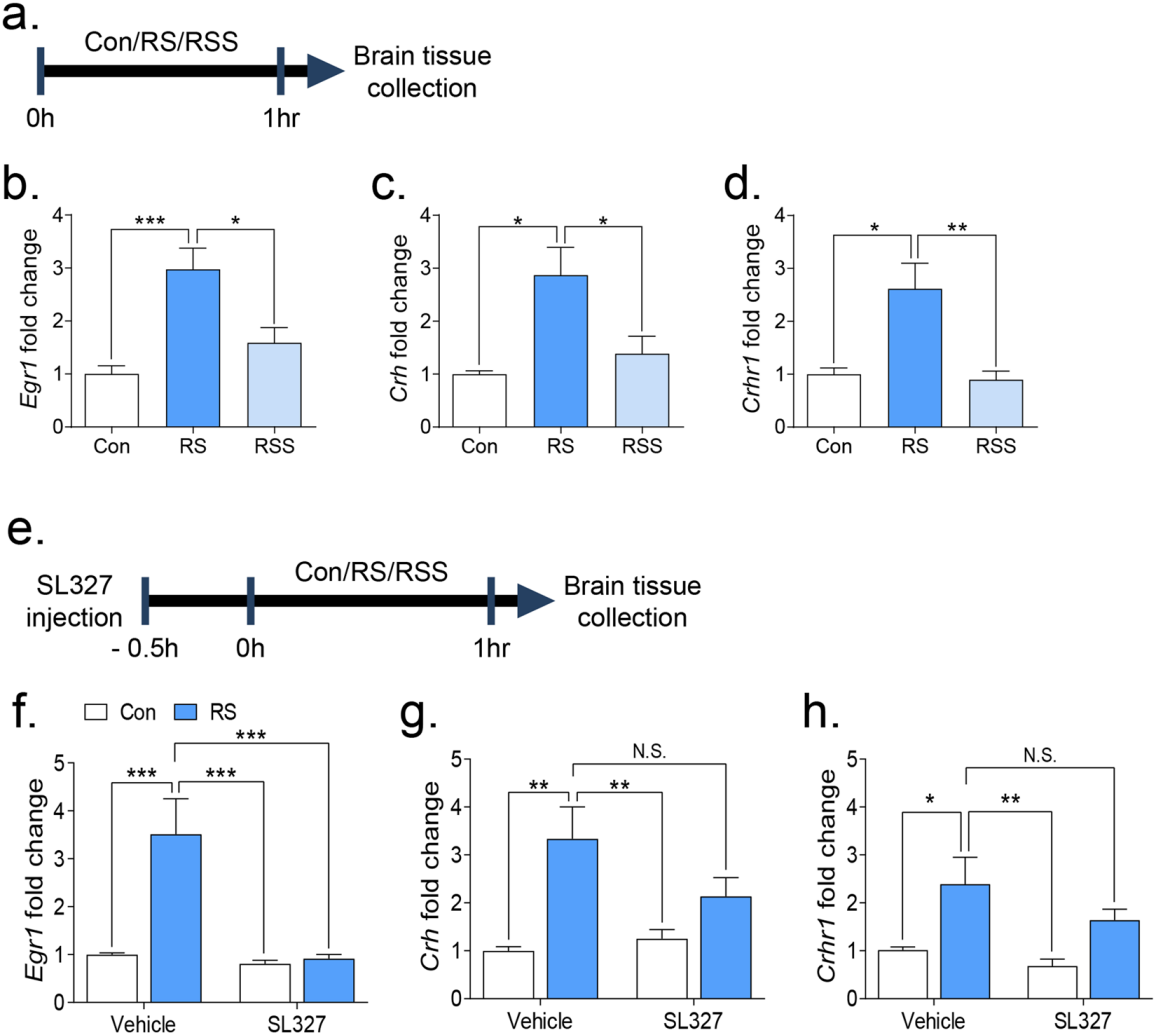
Transcription levels of stress-related genes after restraint stress with social interaction or with the treatment of SL327. Transcription levels of stress-related genes were analysed in the PFC using real-time PCR. (**a**) Scheme of restraint stress followed by brain tissue collection. (**b**–**d**) Gene expression levels of relevant markers in the PFC (n = 7). Statistical analyses were performed using one-way ANOVA followed by Bonferroni’s multiple comparison post hoc tests or Kruskal-Wallis test followed by Dunn’s multiple comparisons post hoc test. (**e**) Scheme of SL327 injection, restraint stress, and brain tissue collection. (**f**–**h**) Gene expression level of relevant markers in the PFC with SL327 treatment (n = 8). Statistical analyses were performed using two-way ANOVA and Bonferroni’s multiple comparison post hoc analysis. Quantifications of each gene expression levels were presented as the fold change from the control value of 1. * is *p* < 0.05, ** is *p* < 0.01, *** is *p* < 0.001. N.S.: no significance, Con: vehicle control group, RS: restraint stress group.

To further identify the underlying mechanisms of the stress-buffering effects of social support, we investigated the mRNA expression levels of previously defined stress-related genes (*Egr1*, *Crh*, *Crhr1*, *Nr3c1*, and *Nr3c2*)^33,34^. We evaluated the transcription levels of candidate genes using real-time PCR in two experimental paradigms (Experiment 1 and Experiment 2, Fig. 5a and e, respectively). We confirmed that restraint stress significantly upregulated the transcription levels of *Egr1*, *Crh*, and *Crhr1* in both experiments 1 and 2 (Fig. 5) but no changes were observed in *Nr3c1* and *Nr3c2* (Supplementary Fig. 4). Remarkably, social interaction and ERK1/2 differentially modulated the transcription levels of those stress-related candidate genes. The presence of a conspecific mouse decreased the previously upregulated transcription levels of *Egr1*, *Crh*, and *Crhr1* in restraint-stressed mice (Fig. 5b: F(2, 18) = 11.55, *p* < 0.001, c: H = 8.045, *p* < 0.05, d: H = 11.04, *p* < 0.01). However, SL327 administration only normalized the *Egr1* transcription levels (Fig. 5f: Restraint × Drug effect: F(1, 28) = 10.31, *p* < 0.01, Restraint effect: F(1, 28) = 12.20, *p* = 0.0016, Drug effect: F(1, 28) = 13.88, *p* < 0.001) with only decreasing trends in *Crh* and *Crhr1* transcription levels (Fig. 5g and h). These results suggest that the ERK1/2 pathway might be part of the overall effects of social support and modulation of *Egr1* mRNA possibly by ERK1/phosphorylation might be involved as a molecular target underlying the stress-buffering effects of social support.

## Discussion

Stress response can be critical for neurological development during the adolescent period^4–6^. Thus, proper strategies to relieve the stress response or  develop resilience against stress should be required. Here, we investigated the role of social support in the stress-induced cognitive impairments in adolescent mice. In this study, we demonstrated that social interaction can alleviate the stress-induced working memory and recognition memory impairments. Additionally, social support can relieve the increased ERK1/2 phosphorylation and plasma corticosterone induced by RS. Lastly, ERK1/2 phosphorylation can be the target molecule to modulate the stress-induced working memory deficits and aberrant transcription of genes. Taken together, our results provide insights in understanding the molecular basis of social support against the negative effects of stress in cognition during the adolescent period.

Here, we employed a pro-social behaviour paradigm and a restraint stress model to evaluate the effects of social support on acute stress at the cellular and behavioural levels^30,35^. Pro-social behaviour is an altruistic attitude that is inclined to help others willingly and has been reported as an innate trait of rodents^30,36^. In this paradigm, the mouse outside the restraint displayed attempts to free the restrained mouse and made frequent nose-to-nose interactions. Our results indicate that the sympathetic behaviour of conspecific mouse helped the restrained mouse overcome the stress-induced various cognitive impairments (Figs 1 and 2), suggesting that social support during stressful conditions can relieve its negative effects. Since previous studies mainly focused on the social support effects after the stressful condition^17,18,37,38^, our results provide an evidence on the importance of continuous social support to gain resilience against stress. This simple model can be a promising tool to study the influence of social support on stress.

In our results, 1 h acute restraint stress reduced the spontaneous alteration percentage in the Y maze test, which measured the spatial working memory and the preference to explore novel environments. Interestingly, the reduced spontaneous alternation was normalized by the presence of social support during restraint stress (Fig. 2b). To ensure that the influence of novelty is not a significant factor to alleviate the effect of RS, we performed a control experiment wherein we put a novel object (stacked Lego blocks) within the visual field of the mouse during RS (Supplementary Fig. 1). In the results, we confirmed that the presence of novel object during the RS is not enough to rescue impaired performance in the Y-maze test (Supplementary Fig. 1b). Thus, we can more confidently say that the converging effects of stranger mouse presence and social interaction (social support) during stress is suggestively alleviating the cognitive consequences of RS. Importantly, given the change of ERK1/2 by RS and RSS, we can hypothesize that more than a certain amount of time is required to produce stress-relieving effects by social support. In the time-dependent ERK1/2 phosphorylation changes by RS (Fig. 3b), the ERK1/2 level was significantly increased at 30 min in both RS and RSS groups versus control group, but the increased ERK1/2 level was significantly normalized to control level after 1 h of RSS. Consistently, 1 h of social support also relieved the stress-induced cognitive impairments and stress-hormonal changes (Figs 2 and 3). Collectively, the duration of social support is an important factor to be considered to produce the stress-buffering effects.

We showed that ERK1/2 mediated the stress-induced working memory deficits in the Y-maze test. ERK1/2 signalling is known to be activated in the PFC, hippocampus, and amygdala during acute stressful conditions^22–24^. Since the PFC is known to be a crucial region for the Y-maze and the NORT performance^39^, while the hippocampus is somewhat equivocal^40–42^, the ERK1/2 changes in these brain regions might be the reason for the impaired performance in the Y-maze, and probably in NORT. Indeed, we identified that ERK1/2 phosphorylation was increased in both the PFC and the hippocampus following the exposure to acute restraint stress, and, interestingly, the increased ERK1/2 phosphorylation was normalized by the presence of a conspecific mouse. Additionally, SL327 administration prior to restraint stress rescued the working memory deficits and blocked the increase of ERK1/2 phosphorylation in the PFC (Fig. 4). Although ERK1/2 activation is required for cognitive processes and neural plasticity changes^31^, an overly activated ERK1/2 can affect cognition including working memory and long-term memory^43^. For example, highly activated ERK1/2 are observed in RASopathies, which are developmental disorders with germ-line mutations in genes encoding proteins for Ras and mitogen-activated protein kinase pathways^44^. Accordingly, patients and animal models of RASopathies usually manifest severe cognitive impairments in working memory and long-term memory processes^43,45–47^, while several reports suggested that ERK1/2 inhibitors can rescue those impairments^47–50^. These lines of evidence support the possible role of ERK1/2 in cognition and neural plasticity changes induced by stress.

Of note, SL327 did not affect the working memory in control mice. Several lines of evidence also suggest that the inhibition of ERK1/2 itself does not affect working memory and short-term memory formation. For example, the ERK1 KO mice did not show any impairment in short-term as well as long-term memory processing^51–53^ but rather showed enhanced learning behaviours and neural plasticity^52^, while ERK2 conditional KO mice performed normally in the Y-maze test^54^ where working memory is important^55^. These findings suggest that ERK-induced regulation of memory process is complex and experimental context-dependent phenomenon, which might explain why control mice given SL327 possibly did not show working memory impairment in the Y maze test.

In the NORT, we showed that restraint stress impaired the novel object recognition of mice in both short- and long-term memory paradigms, particularly during the consolidation and retrieval processes of learning and memory as previously reported^27^. In the short-term memory paradigm (Fig. 2a), restraint-stressed mice displayed decreased total exploration time to both familiar and novel objects (Fig. 2b). One possible factor to consider is the anhedonia-like symptoms that can be induced by the preceding restraint stress^25,56^. However, the anxiety level or locomotor activity of mice were not affected by restraint stress in our study (Supplementary Fig. 2), indicating that anxiety is not the cause of the decreased object exploration. Another factor to consider is that since the animals were allowed to explore the NORT arena immediately after restraint stress, the motivation to explore new objects could be directly affected^57^. Nevertheless, the presence of social support in restraint-stressed mice recovered their recognition ability in the short-term memory paradigm. Furthermore, social support can also relieve the long-term memory consolidation and retrieval impairments caused by RS. These results put forward that social support can prevent the stress-induced impaired recognition in short and long-term memory paradigms.

Along with the improved cognition deficits, we showed that the increased plasma corticosterone level in the RS group was normalized by social support, which can be considered as another important factor for the stress-buffering effects of social support (Fig. 3d). Indeed, the activation of glucocorticoids in the medial prefrontal cortex is known to impair the working memory^58^ possibly via activating the dopamine efflux in the prefrontal cortex^59^. In addition, previous studies demonstrated that stress hormone impairs memory retrieval processes^60^, consistent with our results. Thus, the level of corticosterone is possibly involved in the recovered cognition of RS mice through social support. However, it is difficult to attribute the recovered memory consolidation to the normalized corticosterone levels since stress-related hormones are known to improve memory consolidation^58,61^. Nevertheless, in line with our results, other reports also showed impaired memory consolidation induced by RS in the NORT^27,62^, suggesting that stress hormone is not the sole factor to affect memory consolidation while other factors such as the type of stress, intensity, duration and so on could be playing roles in affecting the memory consolidation processes. To address this question, a more specific study focusing on the machinery of learning and memory will be required.

In our study, acute restraint stress also upregulated the *Egr1*, *Crh*, and *Crhr1* mRNA expressions (Fig. 4) without affecting the glucocorticoid receptor (*Nr3c1*) and mineralocorticoid receptor (*Nr3c2*) expressing genes (Supplementary Fig. 2). Remarkably, the presence of social support alleviated all these transcriptional changes induced by restraint stress, but the administration of SL327 only blocked *Egr1* mRNA expression levels. Thus, it is plausible that social support may have broad effects on the molecular signalling changes in the brain, and ERK1/2 phosphorylation may be one of its downstream signalling molecules. Indeed, a previous study showed that the activation of glucocorticoid receptors induced EGR1 expression in an ERK1/2-dependent manner^34^. Given that EGR1 is an immediate early gene, which is rapidly transcribed and translated^63^, increased *Egr1* mRNA possibly upregulates EGR1 protein expression and modulates the stress-induced synaptic plasticity changes^34,64^. Stress-induced transcriptional change is also part of an adaptation process for the next stress stimuli^21^, which indicates that the normalized transcriptional changes by social support would indirectly represent the relieved stress responses. In this sense, the normalization of *Crh*, *Crhr1*, and *Egr1* mRNA levels by social support may be involved in the stress-induced cognitive impairments. Previously, it was shown that acute stress upregulated the *Crh* and *Crhr1* mRNA expressions, while the deletion of *Crhr1* rescued the acute stress-induced cognitive dysfunction^65^. Thus, further study elucidating the role of normalized genes would be another next step to understand the effects of social support on stress-induced synaptic plasticity changes.

In our study, we demonstrated that social interaction could alleviate the stress-induced cognitive impairments in mice partly by modulating ERK1/2 phosphorylation. Our findings further revealed that ERK1/2 phosphorylation in the prefrontal cortex could have a connection in the stress-buffering effects of social interaction via *Egr1* as a downstream regulator. Although more questions remained to be answered to fully understand the underlying mechanisms behind the stress-buffering effects of social interaction, we believe that the present study will provide novel insights into the signalling pathways linked to social interaction and higher cognitive functions.

## Materials and Methods

### Animals

ICR male mice at 3 weeks old were purchased from Orient Bio (Seoul, Korea) and were habituated for a week in the animal facility before commencing the experiments. They were maintained on a 12-h dark/light cycle (lights off at 2:00 p.m./on at 2:00 a.m.) at controlled temperatures (22 ± 3 °C) and humidity (50 ± 20%). During the habituation period, all animals were acclimated to handling once a day for 1 min for each mouse. Mice were housed at a maximum of six per cage (200 × 260 × 130 mm) at postnatal day 23 (P23) with free access to food and water. Mice from ages 4 to 6 weeks were used for this study and all experiments were performed during the nocturnal period (from 2:00 p.m. to 9:00 p.m.). All procedures related to animal treatments including anaesthesia, euthanasia, and administration were carried out in accordance with the Principles of Laboratory Animal Care (NIH publication No. 85–23, revised 1985) and were approved by the Animal Care and Use Committee of Konkuk University, Korea (KU13156).

### Experiment 1: Restraint stress with social interaction

The mouse restraint is made of transparent Plexiglas with a semi-cylindrical shape and a flat base with tiny holes in the front, back, top and bottom sides and a blocker at the rear part of the animal. Subject mice were randomly assigned to either control group (Con), acute restraint stress group (RS), or acute restraint stress group interacted with a conspecific stranger mouse (RSS) (Fig. 1a). The conspecific mice were of the same age and sex as the subject mice and both have no prior contact when the experiments were performed. Behaviour experiments were performed at P30 through P35. The control group was undisturbed until behaviour tests or brain preparations. The RS and RSS groups were restrained for 10 min, 30 min or 1 h in a new cage prior to brain preparations or restrained for 1 h prior to behaviour studies (Fig. 1b,c). The brain regions were collected as soon as the restraint stress or the restraint stress with social interaction sessions were over.

### Experiment 2: Restraint stress with SL327 pre-treatment

SL327 (APExBIO, A1894) at 30 mg/kg dosage was administered 30 min before inducing restraint stress in mice. The dosage was chosen based on preliminary studies and previous reports^47,66^. SL327 was dissolved in a mixed solution (5%v/v DMSO +1%w/v Pluronic^®^ F-127 (Sigma-Aldrich, P2443) in 0.9%w/v saline). The mixed solution being used as a vehicle in the control group did not cause any aberrant symptoms such as sedation, abnormal gait, salivation, convulsion, tremor, and diarrhoea. After 1 h of acute restraint stress, the mice were sacrificed immediately for brain preparations or subjected to behavioural experiments (Fig. 1d).

### Y-Maze Test

The Y-maze test apparatus is made up of polyvinyl chloride that forms a Y shape with equal arm lengths (35 cm), sidewall height (10 cm), and width (5 cm). The mice were introduced to arm A to start the trial and allowed to freely visit all arms for 8 min. The number of arm entries and the number of triads were scored by an observer “blind” to the test conditions while the movements of subjects were recorded in the CCD camera-assisted motion tracking apparatus and software (Etho-Vision 3.1, Noldus Information Technology, The Netherlands). An entry is considered when all four limbs are inside a specific arm. The percentage of spontaneous alternation was calculated as the number of actual alternations divided by the possible perfect alternations per mouse and multiplied by 100.

### Novel object recognition test (NORT)

We performed the NORT to check whether acute restraint stress can affect the recognition memory when applied during the consolidation and retrieval stages of learning and memory processing^27^, and whether social support can alleviate this effect. The test was done with habituation, familiarization and novel object recognition phases as described previously with some modifications^67^. Subjects were habituated for 10 min a day before the test in the test box made of Plexiglas (40 × 25 × 18 cm). During the familiarization phase, two identical objects were placed in the opposite corners of the test box and the subject mice were allowed to explore for 10 min. There were three ways based on timing that we performed the RS or RSS and the subsequent object recognition phase. The first two methods were done to assess the effect of RS at the consolidation stage of memory processing and involved the application of RS right after the familiarization phase and differed on the inter-trial interval between familiarization and recognition; first with short ITI (test phase right after RS and 1 h after familiarization) and second with long ITI (24 h after familiarization). The third method evaluated the effect of RS at the retrieval stage of memory processing. After the familiarization phase, the mice were kept back to their home cages and 23 h later, RS or RSS was employed. Then, recognition test was done right after the RS. The control mice were returned to their home cage after familiarization and were kept undisturbed until the 5-minute novel object recognition. During the recognition phase, one of the familiar objects was replaced with a new object different in shape and colour. The tests were video-recorded, and the time spent exploring the objects were measured by an observer blind to the group conditions. Object exploration was defined when the mouse was sniffing in close proximity to the object but not when the head was in another direction. The discrimination parameter, calculated as the subtraction of time spent in the novel and familiar objects (T_n_ − T_f_), was also measured. After each trial, the arena floor and the objects were wiped with 70% ethanol to eliminate odour cues for the next subject.

### Brain sampling and Immunoblotting

After stress induction, the prefrontal cortex and the hippocampus of mice brains were rapidly removed and snap-frozen by liquid nitrogen and stored in the deep freezer (−80 °C) until used. Frozen samples were homogenized with 0.5 ml of RIPA buffer (150 mM sodium chloride, 1% Triton X-100, 0.5% sodium deoxycholate, 0.1% SDS, 50 mM Tris, pH 8.0) with Complete Protease Inhibitor Cocktail (Roche, 11697498001), 0.1 mM PMSF, 10 mM NaF, and 1 mM Na_2_VO_4_. Then, samples were centrifuged at 10,000 × g for 30 min and the supernatants were collected and quantified by BCA assay. The quantified supernatants were diluted with 5x SDS-PAGE sample buffer and boiled for 5 min at 95 °C. Equal amounts of proteins for each condition were resolved via electrophoresis on 10% SDS-PAGE gels. The proteins in the gels were transferred to Whatman™ membrane paper for 1.5 h and blocked with 1 ng/ml of polyvinyl alcohol for 1 h. After overnight incubation with anti-ERK1/2 (1:2000, Cell Signalling, 9102), anti-pERK1/2 (1:2000, Cell Signalling, 9101 S), or β-actin (1:50000, Sigma-Aldrich, A5316) in Tris-buffered saline and 0.1% Tween 20 (TBST) with 1% of skim milk, the blots were washed three times with TBST for 10 min each wash. Then, the blots were incubated for two hours with either goat-anti-rabbit (1:4000, Sigma-Aldrich, A0545), or goat-anti-mouse (1:4000, Sigma-Aldrich, A4416) secondary antibody. The blots were again washed three times with TBST and visualized using WEST-ZOL Plus (iNtRON Biotechnology, 16024). Blots were captured using LAS-3000 imaging system (Fuji film) and quantified by multi-gauge V3.0 (Fuji film) while being normalized with the β-actin band.

### Immunohistochemistry

The whole brains from perfused subject mice were fixed with ice-cold 4% paraformaldehyde (Sigma-Aldrich) in PBS for one day after perfusion. The brain samples were dehydrated in 30% sucrose for three days and embedded in the Frozen Section Compound (Leica, 3801480). The frozen samples were sectioned at 40 µm and blocked with 10% Normal Goat Serum (Thermo Fisher Scientific, PCN5000) in 0.025% Tween 20 solution for 1.5 h. The sections underwent washing twice with 0.025% Tween 20 and once with PBS, followed by labeling with anti-pERK1/2 (1:100, Santa Cruz Biotechnology, SC-7383). For immunofluorescence labeling, the sections were incubated with pERK1/2 in 1/3 of 10% Normal Goat Serum for three days at 4 °C. The sections were then rinsed 3 times with 0.025% Tween 20 and twice with PBS followed by incubation with secondary antibody (Alexa Fluor® 488 Goat Anti-Rabbit IgG, 1:500, Thermo Fisher Scientific, A-11008) in 1/3 10% Normal Goat Serum at 4 °C for 2 h. After rinsing twice with PBS, the nuclei in the sections were labelled with TO-PRO3 (1:1000, Thermo Fisher Scientific, T3605) for 10 min, then washed with PBS. The sections were mounted in the coverslip with ProLong® Gold antifade reagent (Thermo Fisher Scientific, P10144). The images were acquired with a fluorescence microscope (Carl Zeiss, LSM710) and assembled in Adobe Photoshop CS6 (Adobe).

### Real-time PCR

Total RNA was extracted from the prefrontal cortices using Trizol reagent and 1 μg of total RNA was reverse transcribed using a RevertAid Reverse transcriptase (Thermo Fisher Scientific, EP0441). Quantitative real-time PCR was performed using ABI7500 (Applied Biosystems) and relative fold changes were calculated using comparative threshold cycle (C_t_) method^68^. Real-time PCR reaction was performed with a mixture of cDNA, primer set, and SYBR® Premix Ex Taq II (Takara, RR820A). The primers used for this study are listed in Table 1.

**Table 1:** Primers sequences of stress-related genes of interest.

Genes	Sequence (F)	Sequence (R)	Source
*Egr1*	aacactttgtggcctgaacc	aggcagaggaagacgatgaa	NC_000084.6^34^
*Crh*	tctcaccttccaccttctgc	ttcctgttgctgtgagcttg	NM_205769.3
*Crhr1*	gccgcctacaactacttcca	ctgcacacagccatcgtact	FJ668671.1
*Nr3c1*	acagactttcggcttctgga	cttctctgtcggggtagcac	NM_008173.3
*Nr3c2*	cagtgcacagtcccatcact	tgaaagaggagagcccacat	NM_001083906.1

### Plasma corticosterone measurement

After an hour of restraint stress (Experiment 1), mice were anesthetized and decapitated to collect the trunk blood. Blood samples were centrifuged at 3000 × g for 10 minutes at 4 °C to obtain the plasma. Each plasma was gently separated from the blood and diluted into one-hundredth volume by using 0.1 M sodium citrate as an anticoagulant. An Enzyme-Linked Immunosorbent Assay (ELISA) was performed to measure the level of corticosterone following the Corticosterone ELISA kit (CAT #. ab108821) from Abcam (Cambridge, United Kingdom).

### Statistical analysis

After performing the Shapiro-Wilk test to check the normality of the data, parametric or nonparametric statistical analysis was performed to find out the statistical significance. If the data satisfies the normality, unpaired t-test was used for paired comparisons, or one-way or two-way analysis of variance (ANOVA) was performed for multiple comparisons followed by Bonferroni’s post-test. For those which did not pass normality, Mann-Whitney test for paired comparisons or Kruskal-Wallis test for multiple comparisons was performed followed by Dunn’s multiple comparison tests as a posthoc test. The comparisons were considered statistically significant when the P value was less than 0.05 (*p* < 0.05). All statistical analyses were conducted using the GraphPad Prism v5 software.

## Electronic supplementary material

Supplementary information

All supplementary datasets

## Data Availability

All data generated or analysed during this study are included in this published article (and its Supplementary Information files).
