# Supplementary material for: Social support rescues acute stress-induced cognitive impairments by modulating ERK1/2 phosphorylation in adolescent mice
Source: Sci Rep. 2018 Aug 13;8:12003. doi: 10.1038/s41598-018-30524-4 (PMC6089908; doi:10.1038/s41598-018-30524-4)
Supplement: Supplementary file 1 — Supplementary information [file 41598_2018_30524_MOESM1_ESM.docx]

**Social support rescues acute stress-induced cognitive impairments by modulating ERK1/2 phosphorylation in adolescent mice**

Ji-Woon Kim^1^†, Mee Jung Ko^1^†, Edson Luck Gonzales^1^†, Ri Jin Kang^1^, Do Gyeong Kim^2^, Yujeong Kim^2^, Hana Seung^1^, Hyun A Oh^1^, Pyeong Hwa Eun^1^, and Chan Young Shin^1,3,*^

^1^ Department of Neuroscience, School of Medicine, Konkuk University, Seoul 143-701, Korea

^2^ Department of Advanced Translational Medicine, Konkuk University, Seoul 143-701, Korea

^3^ Department of Pharmacology, School of Medicine, Konkuk University, Seoul 143-701, Korea

†These authors contributed equally to this work

*To whom correspondence should be addressed

Chan Young Shin, Ph.D.

Department of Pharmacology, School of Medicine, Konkuk University,

1 Hwayang-Dong, Gwangjin-Gu, Seoul 143-701, Korea

Tel) 82-2-2030-7834; Email) [chanyshin@kku.ac.kr](mailto:chanyshin@kku.ac.kr)

**Supplementary information**

**Open field test**

The locomotor activity was assessed in an open field box (40 x 40 x 30 cm). Subject mice were promptly introduced into the arena after stress stimulation and allowed to explore for 20 min. The total distance moved was measured through the CCD camera-assisted EthoVision software (Noldus Information Technology, The Netherlands).

**Elevated plus maze**

The elevated plus maze is composed of two open arms (30 x 5 cm), two closed arms (30 x 5 x 15 cm) and a central (5 x 5 cm) area with 50cm height. The movement of mice and the time spent in the arms were measured for 8 min using EthoVision software (Etho-Vision 3.1, Noldus Information Technology, The Netherlands). In between trials, the arenas were wiped with a 70% ethyl alcohol solution and allowed to dry completely. For further analysis, the duration or distance moved index (open arms/closed arms) was evaluated.

**Supplementary figure 1. The presence of a novel object did not rescue the reduced spontaneous alternation induced by restraint stress**

To ensure that the influence of social interaction is a significant factor to alleviate the effect of RS, we performed a control experiment wherein we put a novel object (stacked Lego blocks) within the visual field of the mouse during RS. (a) Total arm entries and (b) spontaneous alternations in the Y maze test (n=18). All data are expressed as the mean ± S.E.M using bar graphs. One-way ANOVA was performed followed by Bonferroni’s post hoc comparisons. * is *p* < 0.05, *** is *p* < 0.001. Con: vehicle control group, RS: restraint stress group, RSO: restraint stress with novel object group.

**Supplementary figure 2. Acute restraint stress did not change the anxiety level and locomotor activity of mice.**

Elevated plus maze test measuring the open/close arms (a) stay duration ratio and (b) distance moved ratio. The locomotor activity was assessed in an open field box for 20 min (n=10). The (c) total distance moved and the (d) center/total distance moved were measured through the CCD camera-assisted EthoVision software (Noldus Information Technology, The Netherlands). All data are expressed as the mean ± S.E.M. One-way ANOVA was performed followed by Bonferroni’s post hoc comparisons. N.S.: no significance. Con: vehicle control group, RS: restraint stress group, RSS: restraint stress with social interaction group.

Supplementary figure 3. Social interactions reduced the acute restraint stress-induced ERK1/2 phosphorylation in the hippocampus but not amygdala

The activation of ERK1/2 was measured in the hippocampus and amygdala after 10 min, 30 min and 1 h of acute restraint stress. Western blot representative images cropped from separate sets of gels per time point and the corresponding quantitative graphs of ERK1/2-phosphorylation in the (a) hippocampus and (b) amygdala. Quantifications of pERK1/2 bands were normalized by ERK1/2 bands intensity and presented as the fold change from the control value of 1. ERK1/2 phosphorylation was significantly increased in the hippocampus after 1 h of restraint stress (*, *p*<0.05), which was normalized by the presence of a conspecific mouse (**, *p*<0.01). (b) Any significant pERK1/2 changes were not observed in the amygdala at any time point we tested. All data are expressed as the mean ± S.E.M (hippocampus: n= 4 for 10 min, 5 for 30 min and 5 for 1 h; amygdala: n = 4 for 10 and 30 min and n = 7-8 for 1 h). All statistical analyses were performed using one-way ANOVA and Bonferroni’s multiple comparison post hoc analysis. Con: vehicle control group, RS: restraint stress group, RSS: restraint stress with social interaction group.


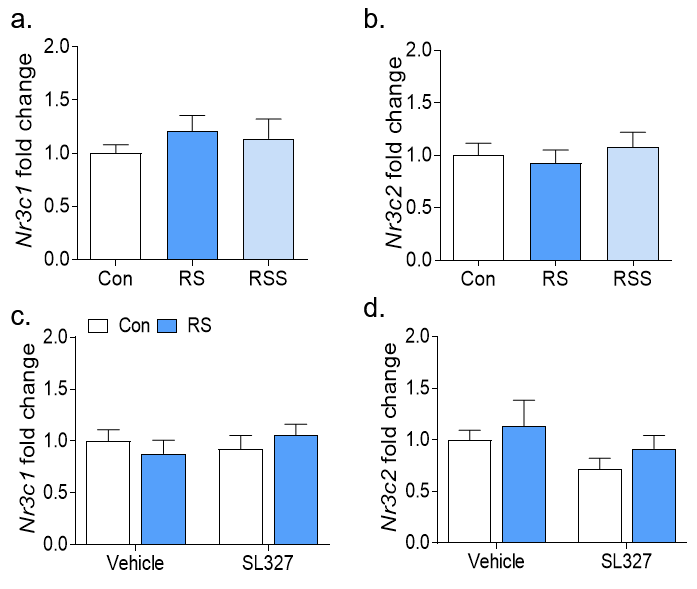


**Supplementary figure 4. mRNA expression levels of other stress-related genes in RS, RSS or RS+SL327 groups**

Levels of *Nr3c1* and *Nr3c2* mRNA expression were analyzed in the PFC using real-time PCR. (a-b) Gene expression levels after RS or RSS (n=7). (c-d) Gene expression levels in RS and RS+SL327 treatment (n=8). Quantifications of each gene expression levels were presented as the fold change from the control value of 1. Statistical analyses were performed using two-way ANOVA and Bonferroni’s multiple comparison post hoc analysis. No significance was noted. Con: vehicle control group, RS: restraint stress group.
